# Helical Virus Structure: The Case of the Rhabdovirus Bullet

**DOI:** 10.3390/v2040995

**Published:** 2010-04-12

**Authors:** Jay C. Brown, William W. Newcomb, Gail W. Wertz

**Affiliations:** 1 Department of Microbiology, University of Virginia School of Medicine, Charlottesville, VA 22908, USA; 2 Department of Pathology, University of Virginia School of Medicine, Charlottesville, VA 22908, USA

## Abstract

Commentary on Ge, P.; Tsao, J.; Schein, S.; Green, T.J.; Luo, M.; Zhou, Z.H. Cryo-EM model of the bullet-shaped vesicular stomatitis virus. *Science* **2010**, *327*, 689–693.

Most viruses are either helical or icosahedral in structure. The two highly symmetric shapes permit viruses to use the same component protein multiple times to create large structures from a minimum number of distinct protein species. The strategy conserves the amount of genetic material viruses need to encode structural proteins.

Although the two basic shapes serve the needs of viruses more or less equally well, structural biologists have had a much easier time determining the structures of the icosahedra. For example, while more than one hundred high resolution structures of icosahedral viruses are now available, the number of comparable helical virus structures is limited to helical plant viruses such as tobacco mosaic virus and filamentous bacteriophage such as *E. coli* phage f1.

It’s not as though helical animal and human viruses are of limited interest. Just the opposite. They include influenza virus plus members of the paramyxo-, rhabdo- bunya-, corona, filo- and arenavirus families, all of which contain important human pathogens. The problem is that structural analysis of these viruses is unusually difficult. The protein-RNA complex is often disordered or weakly ordered in the virion, and the viruses have a membrane, a structure that complicates both crystallization and electron microscopic analysis.

To advance our knowledge of helical virus structure, investigators have focused their attention on the rhabdoviruses, a family of bullet-shaped viruses that includes rabies and vesicular stomatitis viruses (VSV). Rhabdoviruses have a helical nucleocapsid that is well ordered over most of the virion length. Although a membrane is present, it is tightly wrapped around the nucleocapsid, and does not obscure the helix in electron micrographs of the virion (see images of VSV in Figures 1a and 1b). With such excellent images, one would think it would be a simple matter to compute a three-dimensional reconstruction by standard Fourier-Bessel-based methods. No structure has been forthcoming, however, despite the best efforts of many highly talented structural biologists—until now.

Using electron micrographs of VSV preserved in the frozen-hydrated state and a real space reconstruction method, Ge *et al*. [1] have computed the VSV structure at 10.6Å resolution. The results are wonderful. The structure shows a wealth of detail about the nucleocapsid helix, interaction of the nucleocapsid protein (N) with the overlying layer of M protein and contacts between M and the membrane glycoprotein. Unique features at the virion ends are described and there are implications for rhabdovirus assembly. Here we briefly discuss the methods used to compute the reconstruction, the new features revealed about VSV morphology and what this singular accomplishment may mean for future analyses of helical virus structures.

## Prior knowledge of VSV composition and assembly [2]

VSV is a typical rhabdovirus with a bullet shape, a length of ∼190 nm and a diameter of 85 nm. At the core of the virion is the minus sense ssRNA genome bound to N protein. The RNA is 11,161 nucleotides in length, and it is tightly but non-covalently attached to N protein (MW 47.5 kDa; 422 amino acids) creating the nucleocapsid. Each N molecule encapsidates nine nucleotides of the RNA with 1240 N molecules expected in the overall helical structure. In the intact virion, the nucleocapsid helix is organized into ∼35 helical turns with ∼38 N protein subunits/turn in the trunk region (but smaller numbers in the domed end).

The entire nucleocapsid is enclosed in a mono-molecular layer of the matrix protein, M (29 kDa; 229 amino acids; 1826 molecules/virion). M protein is thought to create the precise structure of the nucleocapsid helix in the virion as the nucleocapsid is un-structured in the absence of M [3]. In addition to wrapping the nucleocapsid helix, M also makes contact with the virion envelope membrane. Embedded within the membrane are ∼400 trimers of the virion glycoprotein required for entry of the virus into a host cell.

**Figure 1.**
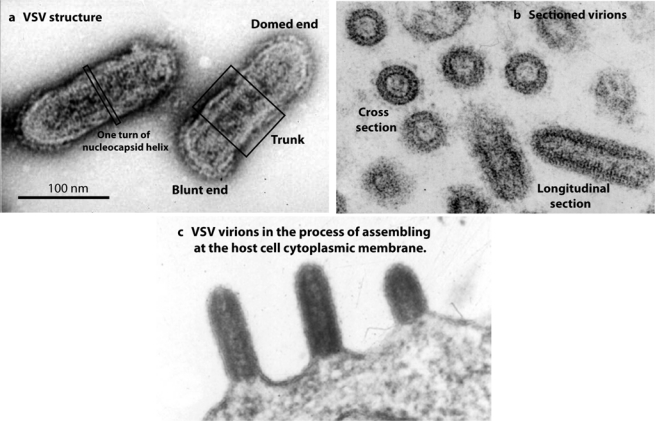
Electron micrographs illustrating VSV structure and assembly. **(a)** and **(b)** show virions in negative stained and thin section preparations, respectively. Note that individual turns of the nucleocapsid helix can be seen in both cases. **(c)** shows virions in the process of assembling at the host cell cytoplasmic membrane. Note that nascent virions bud beginning at the domed end.

VSV assembles by budding at the host cell cytoplasmic membrane. Assembly is initiated by interaction of the nucleocapsid with a specialized region of membrane containing M and G proteins [4]. M and the membrane then bind to the nucleocapsid progressively creating helical turns beginning at the domed virion end. As helical turns are created, the overall structure projects progressively further outward from the host cell (Figure 1c). Assembly is terminated with formation of the blunt end and detachment of the complete virion from the host cell.

## Method of reconstruction

**Figure 2.**
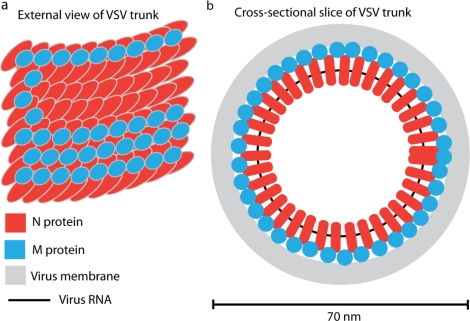
Interpretive drawings illustrating the new VSV structure. **(a)** shows an external view of the N (red) and M (blue) helices as they are found in the trunk region of the mature virion. A portion of the M helix has been removed to illustrate the underlying N helix. The membrane and the glycoproteins are not illustrated. Note that N molecules in one helical turn lie between two N molecules in the turn above. Note also that: (i) the M helix lies between turns of the N helix linking the two N turns together; (ii) there is one M molecule for each N; (iii) M molecules make lateral connections with each other providing stability to the overall structure; and (iii) N protein molecules are tilted slightly upward (27°) with respect to horizontal plane. **(b)** shows a cross-sectional view of the virion in the trunk region. Note that there are 37.5 N molecules in each helical turn and N protein molecules are connected externally by M and in the middle by the virus RNA (black).

Ge *et al.* [1] determined the VSV structure beginning with cryo-electron micrographs of virions imaged at a magnification of 98,000X. Reconstruction focused on the virion trunk, and was computed in two steps. First, the helical parameters were determined by: (a) measuring the pitch of the helix from layer lines in the Fourier transform of the trunk; and (b) two-dimensional classification of trunk images to determine the number of N protein molecules per helical turn. The latter method demonstrated that each N molecule is located between two others in the turn above and in the turn below, an observation showing there are two helical turns in each repeat unit (see Figure 2a). The exact number of N molecules per turn was determined by testing candidate numbers in the reconstruction as described below.

Second, the reconstruction was computed using the iterative helical real space reconstruction (IHRSR) method developed by our colleague Ed Egelman at the University of Virginia [5]. A starting model was created by classifying trunk images according to their mutual similarity. An average of the class averages was then used to center and align individual particles for determination of their rotational angle, the key step in creation of the starting model. Starting angles were identified by the degree of fit with templates calculated at 4° intervals. Angular classes were then averaged, back projected and the helical parameters applied to create an initial model. The initial model was then refined iteratively until it converged on a solution. A total of 644 trunk images were used in the reconstruction reported.

The reliability of the reconstruction was tested by docking two available X-ray crystallographic structures, the N protein-RNA complex [6] and the C-terminal domain of M [7,8]. Both were found to fit well with candidate regions of the cryo-EM volume, a result that authenticates the structure and at the same time allows the N and M proteins to be identified reliably. Docking of the N-RNA complex permitted the directionality of the RNA to be established; the 5′ RNA end was found to be located at the domed end of the virion.

## The structure

VSV structure was shown to consist of two, single, concentric helices, one composed of N protein and RNA (the nucleocapsid) and the other a helically arrayed layer of M. The nucleocapsid helix has outer and inner diameters of 45.0 nm and 30.8 nm, respectively, with layers spaced 5.08 nm apart along the helical axis. There are 37.5 N protein molecules per turn with 75 in the repeating unit (Figure 2b). Each N protein is tilted 27° with respect to the horizontal plane, an un-anticipated feature of the new structure. Individual turns of the nucleocapsid are not tightly bound to each other, a finding that rationalizes the observation that the N-RNA helix is un-structured in the absence of M protein [9].

Each turn of the M protein helix lies in the space between two turns of the nucleocapsid helix making contact with both and holding them together (Figure 2a). There is one M molecule for each N in the overall M protein helix. Individual M protein molecules are found in a U shape with arms called the M-hub and the M-protein-C-terminal domain (Mctd), respectively. The M-hub makes contact with the upper and lower turns of the nucleocapsid helix as illustrated in Figure 2a. The Mctd extends outward and at an angle from the ribonucleocapsid helix in a position to make contact with the virus membrane. Thin projections from the membrane are found to contact the Mctd, and these are interpreted as C-terminal tails from the virus glycoprotein.

In order for rhabdovirus RNA synthesis to take place *in vitro*, virions need to be disrupted with detergent to solubilize the membrane [10]. The new VSV structure suggests disruption may be required to loosen the M protein helix. This would allow the ribonucleocapsid to flex permitting the RNA-dependent RNA polymerase (L protein) to gain access to the template RNA in the nucleocapsid.

A long-standing enigma of rhabdovirus biology is how the viral RNA can function as a template for RNA synthesis despite being tightly bound by N protein. Available evidence indicates that in the nucleocapsid, RNA is resistant to nuclease digestion, even when transcription is in progress [11]. The N structure in complex with RNA shows that N is bi-lobed and that the RNA is sequestered in a 2.0 X 1.0 nm cavity between the two lobes [6]. It is reasonable to expect that the RNA would be protected from nucleases inside the cavity. The nucleocapsid structure, however, does not provide sufficient space to accommodate the large RNA-dependent RNA polymerase molecule. Domain movement to “open” the two lobes of the N protein has been proposed as a means by which the polymerase may gain access to RNA [12]. Removal of the M protein holding the ribonucleocapsid in place may facilitate N domain movement and allow the polymerase to gain access to the RNA template.

## Questions remaining to be answered

The location of the G protein was not revealed in the reconstruction reported. This result was expected, and suggests that the glycoprotein trimers are not arranged with the same helical symmetry as the nucleocapsid-M complex. Further analysis will be required to localize the glycoprotein. Although the new structure accounts for one M protein bound to each N, there is additional M protein in the virion. The experimental copy number for M is 1826 [3], so the number of un-located M molecules is 1826 minus 1240 or 586 molecules, approximately 1/3 of the total M. Possible locations for the missing M protein include the axial channel of the virion and the virion ends, places where M may not be helically arranged.

In addition to N, M and G, VS virions contain P and L proteins, the remaining two proteins encoded in the genome. L is the RNA-dependent RNA polymerase responsible for replication and transcription of the VSV genome while P tethers L to the nucleocapsid. Both proteins are considered to be located in the axial channel of the VS virion, but no candidate features were seen in the current reconstructed volume. There is good evidence for binding of P to the N protein [12], and L is thought to be bound to P. The two may not have been seen in the reconstruction because they are not localized at the same place in each virion or perhaps because of the low copy numbers of L (60/virion) and P (466/virion). Localizing L and P is an important, but challenging problem for future studies.

## Future prospects

In view of the success with VSV, one can expect that the methods employed by Ge *et al.* [1] will soon be focused on other helical viruses. Filoviruses such as Ebola virus are attractive candidates because of their extended helical regions. Domains of ordered nucleocapsid are often seen in influenza virus, and these suggest themselves as candidates for reconstruction. Other possibilities include the paramyxo-, bunya-, corona- and arena- viruses mentioned above.

Like other important structural advances, the work reported here makes very specific predictions about the amino acid sequences involved in protein-protein contacts. M-N, M-M and M-G contacts are examples. Such predictions are amenable to experimental testing with mutants, and one can anticipate that relevant mutational analyses will be forthcoming; they have the potential to authenticate the structure and add to its usefulness. In fact, a start on this effort is reported by Ge *et al.* [1].

For now it is worth taking time to enjoy the new VSV structure and to congratulate the authors for their skill and ingenuity in computing it. Such reflection may allow us to hope that at last we have the tools to make further progress into understanding the structures of helical viruses.
